# Impact of deprivation on breast cancer survival among women eligible for mammographic screening in the West Midlands (UK) and New South Wales (Australia): Women diagnosed 1997–2006

**DOI:** 10.1002/ijc.29983

**Published:** 2016-02-13

**Authors:** Laura M. Woods, Bernard Rachet, Dianne O'Connell, Gill Lawrence, Michel P. Coleman

**Affiliations:** ^1^ Cancer Research UK Cancer Survival Group, Non‐Communicable Disease Epidemiology Unit, London School of Hygiene and Tropical MedicineKeppel StreetLondonUnited Kingdom; ^2^ Cancer Research Division, Cancer Council NSWKings CrossNew South WalesAustralia; ^3^ Breast Cancer Audit Consultant and Former Director, West Midlands Cancer Intelligence Unit, Public Health Building, University of BirminghamBirminghamEngland

**Keywords:** breast cancer, net survival, New South Wales, West Midlands, England, Australia, deprivation, socioeconomic, cancer screening

## Abstract

Women diagnosed with breast cancer in the UK display marked differences in survival between categories defined by socio‐economic deprivation. Timeliness of diagnosis is one of the possible explanations for these patterns. Women whose cancer is screen‐detected are more likely to be diagnosed at an earlier stage. We examined deprivation and screening‐specific survival in order to evaluate the role of early diagnosis upon deprivation‐specific survival differences in the West Midlands (UK) and New South Wales (Australia). We estimated net survival for women aged 50–65 years at diagnosis and whom had been continuously eligible for screening from the age of 50. Records for 5,628 women in West Midlands (98.5% of those eligible, mean age at diagnosis 53.7 years) and 6,396 women in New South Wales (99.9% of those eligible, mean age at diagnosis 53.8 years). In New South Wales, survival was similar amongst affluent and deprived women, regardless of whether their cancer was screen‐detected or not. In the West Midlands, there were large and persistent differences in survival between affluent and deprived women. Deprivation differences were similar between the screen‐detected and non‐screen detected groups. These differences are unlikely to be solely explained by artefact, or by patient or tumour factors. Further investigations into the timeliness and appropriateness of the treatments received by women with breast cancer across the social spectrum in the UK are warranted.

## Abstract 2

**What's new?**

In the UK, affluent women are known to have higher breast cancer survival than deprived women, regardless of access to screening. In this study, the authors examined net survival by socioeconomic status in the West Midlands (UK) and New South Wales (Australia). They found a persistent ‘deprivation gap’ in survival in the former, but not in the latter region. Further investigation into the treatment and follow‐up received by women with breast cancer across the social spectrum in the UK is warranted.

Despite advances in survival amongst women diagnosed with invasive breast cancer in recent decades, women in the UK display marked differences in survival between categories defined by socioeconomic deprivation.^1^, ^2^, ^3^, ^4^ The survival differentials have narrowed more recently, but more deprived women are still at a considerable disadvantage over affluent women.^3^, ^5^ These inequalities are a key focus of public health policy.^6^

Later diagnosis amongst the more deprived groups, associated with later stage of disease at presentation, is one of the possible explanations for these patterns.^7^ Women whose breast cancer is screen‐detected are usually asymptomatic and are often diagnosed at an earlier stage than women diagnosed following symptomatic presentation. It has previously been shown that deprivation differences in screen‐detected survival are smaller than for those who present symptomatically.^8^ Screening thus provides an opportunity to examine the contributions of the timeliness of diagnosis and stage of disease to socioeconomic differences in survival. In a companion article, we have examined international differences in survival between screened and non‐screened women from West Midlands and New South Wales who had been eligible for screening from their 50th birthday.^9^ We found that net survival was persistently higher in New South Wales than in the West Midlands, among both screen‐detected and non‐screen‐detected women. Here, we examine socioeconomic differences in net survival among the same women, and we compare the patterns observed in Australia and England.

## Materials

The data have been described.^9^ Briefly, the cohort consisted of women diagnosed with an invasive, primary breast cancer at age 50–65 years during the period January 1, 1997 to December 31, 2006 and aged 51 years or younger on 1 January 1997. These very specific eligibility criteria were designed to include only women who had been consistently eligible for population‐based mammographic screening throughout the period 1997 to 2006. The resulting cohort gradually increased over time: the median month of diagnosis was August 2003 in West Midlands and November 2003 in New South Wales. All women were followed up to December 31, 2008. Information was obtained on each woman's age at diagnosis (completed years); the month and year of diagnosis and (if dead) of death; the subsite, morphology, behavior and grade of the tumour, and all information pertaining to the extent of disease at diagnosis. Extent of disease (stage) at diagnosis was coded as localised (confined to the organ of origin), regional (spread to adjacent muscle, organ, fat or connective tissue, or to regional lymph nodes), distant (distant metastasis) or unknown. The data were linked to the population‐based mammographic screening service records in each region to establish each woman's screening status at diagnosis. We defined four groups: 1) women whose cancer was detected at a routine screen, 2) women who presented with cancer following a negative screen but before being invited to their next routine screen (interval cancers), 3) women who presented with cancer after at least one negative screen but who had not attended their most recent appointment (lapsed attenders), and 4) women who presented with cancer clinically and had never attended screening. We compared women in the screen‐detected group (category 1) to all those with non‐screen‐detected cancer (categories 2, 3 and 4).

In each region, all tumour records were linked to an ecologically defined deprivation category based on the quintile of the regional distribution of the percentage of unemployment in their small area of residence. We used unemployment because it is an internationally comparable measure of deprivation and because we have previously shown that differences in survival by deprivation assessed with the unemployment rate are similar to those measured with two highly validated and locally defined deprivation measures.^1^ We used the smallest geographic areas available for the 2001 census to maximise the accuracy of the ecological data in each country. Unemployment rates were calculated for each census district (CD) in New South Wales [mean population 539, standard deviation (s.d.) 254] and each Lower‐Level Super‐Output Area (LSOA, mean 1,513, s.d. 194) in West Midlands. Despite the larger size of LSOAs, we have shown previously that they are comparable to smaller areas of similar size to the CDs when used to examine deprivation differences in cancer survival in England, because they are more socially homogeneous.^10^ Only 17 (0.1%) of records failed to match to a small area; these women were excluded.

## Methods

Our methods have been described in detail.^9^ Briefly, we estimated net survival using the non‐parametric Pohar‐Perme estimator.^11^ We estimated expected survival from regional life tables, derived for each year of follow‐up. To account for the potential effect of lead‐time bias, we calculated additional survival time due to screening, *E*(*s*), for the screen‐detected group.^12^ We applied 10 separate simulations to obtain *E*(*s*)_1_, *E*(*s*)_2_ … *E*(*s*)_10_, assuming that survival times were exponentially distributed with a mean of *E*(*s*). We considered tumours to be over‐diagnosed if they would not have been detected symptomatically during the woman's lifetime or during the study period (before December 31, 2006). For the screen‐detected group, we used the corrected survival times to estimate non‐parametric net survival for each of ten separate data sets. We used the rules established by Rubin^13^ for the recombination of estimates in a multiple‐imputation setting to derive an overall estimate of net survival and its variance, adjusted for lead‐time bias and over‐diagnosis. Where estimates were recombined and no estimate could be given due to small numbers of deaths, we recalculated the estimates using at least five of the simulations. These estimates gave an indication of the trend in survival for groups with small numbers of deaths.

## Results

We analysed data for 5,628 women in West Midlands (98.5% of those eligible, mean age at diagnosis 53.7 years) and 6,396 women in New South Wales (99.9% of those eligible, mean age at diagnosis 53.8 years). We excluded a small number of women (0.7%) who were known to the registry only because breast cancer had been mentioned on their death certificate (DCOs) or if the sequence of dates provided was illogical.

Forty‐four percent of women in New South Wales and 49% in West Midlands resided in affluent localities (deprivation quintiles 1 and 2, Table 1). In West Midlands 13.1% were in the most deprived category compared to 17.0% in New South Wales. There was a similar pattern by stage in each region: more than half of the women were diagnosed with a localised tumour, and more than a third had regional disease at diagnosis. The cancer registry did not have sufficient information to derive stage of disease for 5.3% of women in New South Wales and 8.9% in West Midlands. Ten per cent of the women diagnosed in West Midlands and 7.6% of the women diagnosed in New South Wales died during the period up to December 31, 2008.

**Table 1:** Characteristics of the study cohort: women aged 50–65 (mean age 53.7 years) diagnosed with invasive breast cancer 1997–2006 in New South Wales (Australia) and the West Midlands (UK)

	New South Wales		West Midlands	
	N	%	N	%
**Total number of women**	6,396	100.0	5,628	100.0
**Mean age at diagnosis (years)**	53.8		53.7	
**Deprivation Quintile** a				
1 ‐ Affluent	1,388	21.7	1,455	25.9
2	1,437	22.5	1,294	23.0
3	1,277	20.0	1,110	19.7
4	1,198	18.7	1,024	18.2
5 ‐ Deprived	1,088	17.0	736	13.1
Unknown	8	0.1	9	0.2
**Extent of disease at diagnosis** b				
Localised	3,445	53.9	3,053	54.2
Regional	2,337	36.5	1,953	34.7
Distant	276	4.3	124	2.2
Unstaged	338	5.3	498	8.8
**Vital status** c				
Dead	485	7.6	610	10.8
Alive	5,911	92.4	5,018	89.2

a Quintile of the unemployment rate of the small area of residence at the 2001 census (see text).

b West Midlands data was recoded using the rules applied in New South Wales.^9^

c Follow‐up was complete for all women up to December 31, 2008.

Net survival at 5 years was higher in New South Wales (93.4%) than in West Midlands (90.9%) (Table 2). The absolute difference in the overall five‐year net survival estimates for the two regions was 2.5%, which is consistent with our previous findings for women aged 50–69 diagnosed up to 1999.^1^ Women whose cancer was screen‐detected had higher survival than women whose cancer was not screen‐detected in both regions, even after adjustment for lead‐time bias and over‐diagnosis.

**Table 2:** Deprivation‐ and screening‐specific net survival estimates: women aged 50–65 (mean age 53.7 years) diagnosed with invasive breast cancer 1997–2006 in New South Wales (Australia) and the West Midlands (UK)

	West Midlands			New South Wales		
		Net Survival^a^, % (CI)			Net Survival, % (CI)	
	Number (%)	1‐year	5‐year	Number (%)	1‐year	5‐year
**All**	**5,628 (100.0)**	**97.7 (97.2,98.1)**	**90.9 (89.9,91.7)**	**6,396 (100.0)**	**98.6 (98.3,98.9)**	**93.4 (92.6,94.1)**
Affluent (quintiles 1&2)	2,749 (48.8)	98.2 (97.6,98.7)	92.6 (91.3,93.8)	2,825 (44.2)	98.6 (98.0,99.0)	94.1 (92.9,95.0)
Deprived (quintiles 3,4&5)	2,879 (51.2)	97.2 (96.5,97.8)	89.2 (87.8,90.4)	3,570 (55.8)	98.7 (98.2,99.0)	92.8 (91.7,93.8)
**Screen‐detected**	**2,524 (44.8)**	**99.9 (98.8,100.0)**	**97.5 (96.4,98.3)**	**2,335 (36.5)**	**99.8 (99.2,99.9)**	**98.5 (97.5,99.1)**
Affluent	1,252 (22.2)	99.9 (95.2,100.0)	99.0 (97.2,99.6)	1,068 (16.7)	99.8 (98.7,100.0)	98.7 (96.9,99.4)
*Affluent: corrected* b	*761 (13.5)*	*98.8 (97.9,99.7)*	96.4 (94.4,98.4)*	*625 (9.8)*	99.0 (98.1,99.9)*	96.9 (94.9,98.9)*
Deprived	1,272 (22.6)	99.9 (98.0,100.0)	96.0 (94.2,97.3)	1,267 (19.8)	99.8 (98.8,100.0)	98.4 (96.8,99.2)
*Deprived: corrected* b	*773 (13.7)*	*98.4 (97.5,99.4)*	93.0 (90.6,95.5)*	*765 (12.0)*	*98.8 (98.0,99.7)*	96.3 (94.4,98.3)*
**Non‐screen detected**	**3,104 (55.2)**	**95.9 (95.1,96.6)**	**85.5 (84.1,86.9)**	**4,060 (63.5)**	**97.9 (97.4,98.3)**	**90.4 (89.3,91.5)**
Affluent	1,497 (26.6)	96.8 (95.7,97.6)	87.5 (85.4,89.3)	1,757 (27.5)	97.8 (96.9,98.4)	91.3 (89.7,92.8)
Deprived	1,607 (28.6)	95.1 (93.8,96.0)	83.7 (81.6,85.6)	2,303 (36.0)	98.0 (97.3,98.6)	89.7 (88.1,91.1)

a Net survival estimate at the time of previous event before first or fifth anniversary of diagnosis.

b Cases are excluded due to imputed follow‐up being greater than observed follow‐up (see text). Values are the mean of the 10 imputed data sets with the exception of * which is the mean of at least 5 estimates.

**Figure 1:**
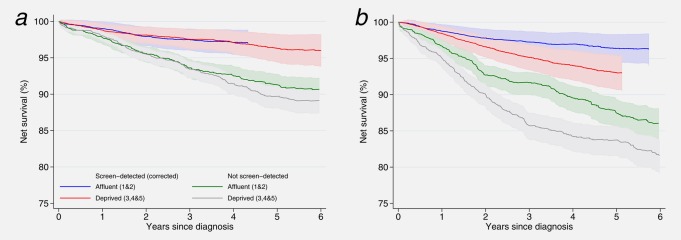
Net survival estimates up to 6 years after diagnosis by deprivation and screening category: women aged 50–65 (mean age 53.7 years) diagnosed 1997–2006 and followed up to December 31, 2008. (*a*) New South Wales, Australia; (*b*) West Midlands, UK.

There was an extremely striking difference between New South Wales and West Midlands in the patterns of survival by screening status and deprivation. In New South Wales, net survival was very similar in both affluent and deprived groups up to 3.5 years after diagnosis, regardless of whether or not the cancer had been screen‐detected (Fig. 1a). In West Midlands, in stark contrast, net survival for the deprived was substantially lower than for the affluent, for women with screen‐detected and non‐screen‐detected cancers, between 1 and 5 years after diagnosis (Fig. 1b). In the first year after diagnosis screen‐detected women in West Midlands had similar survival, irrespective of their deprivation status. Amongst affluent women who had been screen‐detected cancer survival was similar in New South Wales and West Midlands (97% 4 years after diagnosis).

Deprived women in the West Midlands whose cancer was screen‐detected had 5‐year survival 3.3% lower than their counterparts in New South Wales. In fact, their survival was more similar to that of women in New South Wales whose cancer was not detected by screening. The deprivation “gap” in five‐year survival within the West Midlands was around 3.5% in both screening groups, whereas in New South Wales it was much smaller; 1.6% in the non‐screen‐detected group and just 0.6% amongst the screen‐detected group. Five‐year net survival for deprived women with non‐screen detected cancers in the West Midlands was 84%, 6% lower than New South Wales (90%).

**Figure 2:**
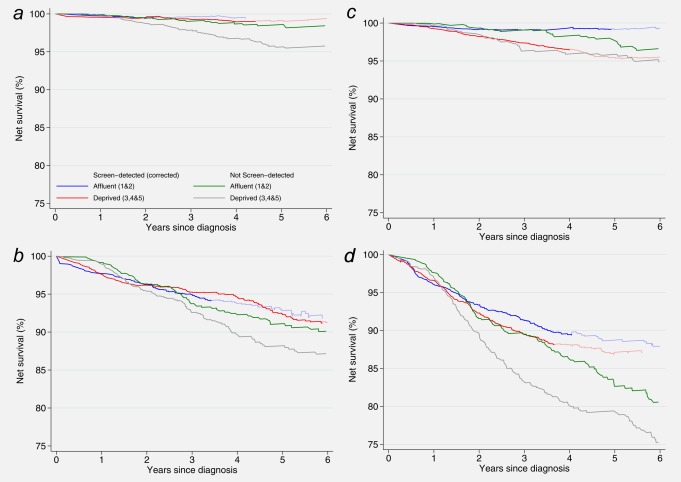
Net survival estimates up to 6 years after diagnosis by deprivation, screening and stage of disease at diagnosis: women aged 50–65 (mean age 53.7 years) diagnosed 1997–2006 and followed up to December 31, 2008. (*a*) New South Wales, localised cancers; (*b*) New South Wales, regional cancers; (*c*) West Midlands, localised cancers; (*d*) West Midlands, regional cancers. Footnote to Figure 2: Corrected survival is in paler colours when based on fewer than 10, but more than 5 recombined estimates (see text).

Similar patterns of survival were observed both amongst women with localised disease and with regional spread. In New South Wales, survival amongst affluent and deprived women was broadly similar for both localised and regional disease, although the survival for deprived women with non‐screen detected cancer was lower than the other groups (Figs. 2a and 2b). Conversely, in the West Midlands deprived women in both screening groups displayed lower survival than affluent women (Figs. 2c and 2d). Deprived women diagnosed in the West Midlands with regional, non‐screen detected disease had the lowest survival: net survival was 80% at four years.

## Discussion

We have compared net survival among women eligible for breast cancer screening who were diagnosed with an invasive cancer between 1997 and 2006 in West Midlands and New South Wales. We have demonstrated stark differences in the pattern of survival by deprivation.

### Deprivation‐specific survival patterns

In New South Wales, survival was broadly similar amongst affluent and deprived women, irrespective of whether their cancer was screen‐detected. There were small survival differences between affluent and deprived women for localised and regional disease. By contrast, in the West Midlands, there were large and persistent differences in survival between deprived and affluent women in both screen‐detected and non‐screen‐detected groups. Survival differences between affluent and deprived women were evident for both localised and regional disease. In the West Midlands, the deprivation “gap” was greatest for women diagnosed symptomatically with regional disease.

Survival amongst affluent women whose cancer was diagnosed via screening was very high (97% 4 years after diagnosis) and very similar in West Midlands and New South Wales. This is the only study to have shown a group of Australian women with similar survival to a group of UK counterparts; previous studies have all shown a large and persistent survival disadvantage for all groups of women diagnosed in the UK.^1^, ^14^, ^15^, ^16^, ^17^ This was, however, the only group of women in which survival was similar.

### Strengths and limitations

This study is unique because international differences in cancer survival between affluent and deprived women have been examined by the individual woman's screening status. We have achieved this via the linkage of each woman's individual cancer registration record to her screening service records in two different countries as well as to the deprivation characteristics of the immediate vicinity of each woman's residential address. We have used a conservative approach, whereby all the women in our study would have received invitations to screening from their 50th birthday. Our results are thus not affected by the inclusion of women only eligible for screening from older ages. We have corrected our results for lead‐time bias and for over‐diagnosis. These biases together lead to apparently better survival in the screen‐detected group when not appropriately considered. We discuss the interpretation of these corrected rates in detail in the companion paper.^9^

There are, of course, limitations to our approach which must be considered. First, we were not able to derive deprivation‐specific life tables for New South Wales, even though these were available in the West Midlands. We conducted a sensitivity analysis to demonstrate the potential impact of this upon our results. We first applied deprivation‐specific life tables to the West Midlands data. We then used the mortality rate ratios between the quintiles of unemployment in West Midlands to estimate plausible deprivation life tables for New South Wales. By doing so, we assumed that the deprivation difference in the underlying risk of death from causes other than breast cancer was the same in the two regions. The use of deprivation life tables reduced the deprivation difference in survival, as would be expected. The reduction was relatively small, between 10 and 12% of the (relative) difference in net survival observed in West Midlands could be attributed to the absence of deprivation‐specific life tables. In New South Wales, where the observed differences were much smaller, the impact of using a deprivation‐specific life table was much less marked, and resulted in almost no difference between the deprivation groups. Thus, the absence of deprivation‐specific life tables in this study does not have a significant impact upon our conclusions, nor explain the differences observed in the West Midlands.

Second, differences in the screening interval in the two regions might potentially explain some of the deprivation‐specific differences in survival. In New South Wales women are invited to be screened every 2 years, whereas in the West Midlands the interval length is 3 years. This implies there may be some residual confounding by stage of disease in the screen‐detected group, since within each stage grouping the tumours detected by screening are likely to be relatively more advanced in West Midlands. However, we observed similar deprivation differences in survival amongst women who were not screen‐detected. Also, we observed the largest deprivation “gap” for regional cancers in West Midlands. Together, these observations suggest that although differences between the two regions for the screen‐detected group may be slightly over‐estimated, this cannot explain the differences observed for the non‐screen‐detected group.

A related concern is the possibility that the accuracy of the staging information is different in each region and in particular between deprivation categories. Poorer quality staging in the West Midlands than in New South Wales (either due to less detailed recording of staging variables or inadequate investigation) would lead to artefactually poorer stage‐specific survival in the West Midlands and/or in the more deprived groups.^18^ We consider that within each region reporting accuracy is unlikely to be biased by deprivation group. It is, however, possible that women in New South Wales and in affluent localities in the West Midlands undergo more extensive staging investigations. No information on the thoroughness of staging investigation (*e.g*., number of nodes examined) was obtained for this study. However, there was no difference in the proportion of women in each deprivation category with microscopically verified cancer in either region, or between regions (over 99.4% were microscopically verified). Missing data on stage of disease did not vary significantly between deprivation categories in New South Wales. Although a greater proportion of women in West Midlands were missing staging information in comparison to New South Wales, it was more frequently missing for the affluent than the more deprived in the West Midlands. This may be related to the treatment of a small proportion of affluent women in the private sector (Lawrence G. Personal Communication: Further analysis of ICBP treatment data v1.2, 2013). We thus have no strong evidence to suggest that thoroughness of staging or accuracy of reporting is a probable explanation for the deprivation patterns observed, but further work would be required to formally assess whether thoroughness of staging varies by socioeconomic status. Residual confounding by stage also remains a possibility, whereby tumours within each staging category are relatively more advanced in deprived groups compared with the affluent groups.

Differences in survival are likely to be in part due to the differences in the way screening is delivered in these two localities. Because mammography is obtained in Australia both through BreastScreen and the Medicare Benefits Scheme (MBS), *de facto* screening is likely to occur to a certain degree, which means that some women in New South Wales recorded in our study as never attenders, were, in fact, screen‐detected. We have shown, however, by a sensitivity analysis in which the proportion of women in the screen‐detected group was modified, that the differences observed are robust to this bias.^9^ Although it is probable that we incorrectly allocated some women to the never‐attender group in New South Wales, information on their personal characteristics and the features of their cancer would not have been compromised since these data items were collected from the Cancer Registry, rather than via the screening service.

The ethnic profile of each population must also be considered. In the Australian setting, it has been shown that breast cancer survival is significantly lower for those of Aboriginal descent.^19^ However, these tumours represent only 1% of breast cancers registered,^20^ so this will have a minimal impact upon our results overall. By contrast, in West Midlands the proportion of non‐White women registered with breast cancer is nearer 4%, but we have shown that, after accounting for deprivation, the survival of the Asian and Black women is not different to that of White women.^21^, ^22^

Overall, survival were high for the cohort of women examined. As a consequence, there were a relatively small number of deaths which precluded modelling the effect of screening status and deprivation on survival simultaneously. Small numbers also prevented accurate estimations of survival for women with distant disease. We therefore regrouped the deprivation indicator into two categories. More extreme deprivation differences would have been observed had we examined all five quintiles of unemployment separately, or if we had used the overall scale as a continuous variable. As such, our results are indicative of a more marked underlying trend. We chose to use a comparable measure of deprivation, the quintile of unemployment, rather than a locally validated score. This was so that we were able to directly compare the results between the regions. The applicability of the proportion of unemployed as a measure of deprivation for women of this age group may be questioned. In the absence of individual data on socioeconomic status from the cancer registry, ecological measures are the next best way to derive estimates by deprivation in the population‐based setting. In our previous work, we have shown that survival estimates are not very sensitive to the score itself^23^ and that when comparing Australia and England the use of a locally derived deprivation measure in each country did not change the conclusions.^1^, ^24^ Since we used, in both settings, the smallest possible geographical area to define the unemployment rate in the vicinity of each women's residential address, we are confident our results are reflective of a true underlying trend of survival by deprivation.

### Possible explanations

Our results show that affluent women presenting via screening have similar survival in both New South Wales and West Midlands, but that differences remain between the more and less deprived in West Midlands and amongst those whose cancer is not screen‐detected. These differences cannot be explained in full by either artefact or by the limitations of our approach.

The fact that screen‐detected, affluent women in both localities have similar survival indicates that these women are being treated with equal effectiveness, that is, similar outcomes are attained in both localities. The fact that survival for the screen‐detected is similar for both deprivation groups during the first year following diagnosis further implies that initial treatment amongst these women is equally effective in the short‐term. The magnitude of the deprivation disadvantage 5 years after diagnosis in the West Midlands was similar for both screen‐detected and non‐screen‐detected groups. This further suggests that the underlying cause of the deprivation‐specific survival differences is unlikely to be simply whether the disease is symptomatic or not, or the mode of the woman's presentation, or even the stage at which the disease is detected.

We have previously conducted an extensive review of the origins of deprivation differences in survival^7^ where we conceptualised three possible groups of explanations for socioeconomic differences; tumour factors (stage, morphology, grade), patient factors (comorbidity, compliance with treatment), and healthcare system factors (timeliness and effectiveness of treatments received). In this study, we have observed deprivation differences for both localised and regional disease. Additionally, these are relatively young women (mean age 54 years) amongst whom significant comorbidities which contraindicate breast cancer treatment are relatively infrequent, (Morris, M: personal communication). We thus consider it more likely that the deprivation‐specific differences in breast cancer survival that we have shown here for the West Midlands are due to the longer‐term effectiveness of the treatment received by affluent women, compared to more deprived women. These might feasibly arise either from healthcare system or patient factors, symptom awareness^25^ including thoroughness of staging leading to timeliness and appropriateness of initial treatments,^26^ variations in specialist treatments,^27^ subsequent clinical follow‐up such as variations in the initiation, adherence and persistence of post‐operative therapies,^28^ or from the complex interaction between two or more of these factors. How and why these might vary so considerably in the UK, where they do not appear to in Australia, may in turn be related to a number of factors, including the way treatment for breast cancer is decided, delivered, received and funded,^27^ and how individuals from different socio‐demographic contexts interact with the healthcare system including those professionals who work within it.^29^ Some research has addressed this question in the context of racial differences in the USA^30^ but research is lacking in the specific context of deprivation differences in the UK.

### Consistency with other studies

Our results for the West Midlands are consistent with a separate analyses of women diagnosed in the same region^8^ and largely consistent with the only other directly comparable analysis on a separate cohort that we could identify. In this, McKenzie *et al*.^31^ have shown a persistent deprivation gap amongst screen‐detected women diagnosed up to 2006 in the South West of England amongst both screen‐detected and non‐screen detected women. In that analysis, however, screening status was missing for 54% of the women. Further, no adjustment was made for lead‐time bias or over‐diagnosis, and thus these results cannot be considered to be reliable. In other similar work outside the UK, the introduction of screening mammography in Italy has been associated with either an attenuation^32^, ^33^ and in the Netherlands an increase^34^ in the differences between socioeconomic groups. None of these studies examined survival by an individual woman's screening status, however, and are thus susceptible to ecological bias.

## Conclusions

We have examined a cohort of young, economically active women, diagnosed with breast cancer in the postscreening era. The vast majority of these women would have been treated with curative intent, and overall estimates of net survival were high. Amongst women resident in West Midlands, we have identified large and persistent differences in survival between affluent and deprived women, for both screen‐detected and non‐screen‐detected groups. These differences are not likely to be explained solely by artefact, or by patient or tumour factors. Further investigations into the timeliness and appropriateness of the treatments, adherence to treatment and, or the clinical follow‐up received by women with breast cancer across the social spectrum are required, and whether more recent improvements in cancer care have influenced these patterns. Studies which address the question of how and why such factors might vary with deprivation status in the UK but not in Australia would also help to understand underlying causes of the deprivation gap in breast cancer survival in the UK.

## References

[1] Woods LM , Rachet B , O'Connell D , et al. Large differences in patterns of breast cancer survival between Australia and England: a comparative study using cancer registry data. Int J Cancer 2009;124:2391–9. 19180628 10.1002/ijc.24233

[2] Coleman MP , Babb P , Damiecki P , et al. Cancer survival trends in England and Wales 1971‐1995: deprivation and NHS Region. Series SMPS No. 61. London: The Stationery Office, 1999.

[3] Coleman MP , Rachet B , Woods LM , et al. Trends and socioeconomic inequalities in cancer survival in England and Wales up to 2001. Br J Cancer 2004;90:1367–73. 15054456 10.1038/sj.bjc.6601696 PMC2409687

[4] Rachet B , Ellis L , Maringe C , et al. Socioeconomic inequalities in cancer survival in England after the NHS cancer plan. Br J Cancer 2010;103:446–53. 20588275 10.1038/sj.bjc.6605752 PMC2939774

[5] Lyratzopoulos G , Barbiere JM , Rachet B , et al. Changes over time in socioeconomic inequalities in breast and rectal cancer survival in England and Wales during a 32‐year period (1973‐2004): the potential role of health care. Ann Oncol 2011;22:1661–6. 21199888 10.1093/annonc/mdq647

[6] Department of Health, Cancer Reform Strategy, 2007 http://www.nhs.uk/NHSEngland/NSF/Documents/Cancer%20Reform%20Strategy.pdf.

[7] Woods LM , Rachet B , Coleman MP. Origins of socio‐economic inequalities in cancer survival: a review. Ann Oncol 2006;17:5–19. 16143594 10.1093/annonc/mdj007

[8] All breast cancers report: a UK analysis of all symptomatic and screen‐detected breast cancers in 2006. NHS Cancer Screening Programmes; West Midlands Cancer Intelligence Unit; National Cancer Intelligence Network, 2009.

[9] Woods LM , Rachet B , O'Connell D , et al. Are international differences in breast cancer survival between Australia and England present amongst both screen‐detected women and non‐screen‐detected women? Survival estimates for women diagnosed in West Midlands and New South Wales 1997‐2006. Int J Cancer, in press. 10.1002/ijc.29984 PMC4788140 26756306

[10] Woods LM , Rachet B , Riga M , et al. Geographical variation in life expectancy at birth in England and Wales is largely explained by deprivation. J Epid Comm Health 2005;59:115–20. 10.1136/jech.2003.013003 PMC1733001 15650142

[11] Pohar‐Perme M , Stare J , Estève J. On Estimation in Relative Survival. Biometrics 2012;68:113–20. 21689081 10.1111/j.1541-0420.2011.01640.x

[12] Duffy SW , Nagtegaal ID , Wallis M , et al. Correcting for lead time and length bias in estimating the effect of screen detection on cancer survival. AmJ Epidemiol 2008;168:98–104. 18504245 10.1093/aje/kwn120

[13] Rubin DB. Multiple imputation for non‐response in surveys. New York: Wiley, 1987.

[14] Yu XQ , O'Connell DL , Forman D. Comparison of cancer survival in UK and Australia: rates are higher in Australia for three major sites. Br J Cancer 2004;91:1663–5. 15477869 10.1038/sj.bjc.6602154 PMC2409957

[15] Coleman MP , Forman D , Bryant H , et al. Cancer survival in Australia, Canada, Denmark, Norway, Sweden, and the UK, 1995‐2007 (the International Cancer Benchmarking Partnership): an analysis of population‐based cancer registry data. Lancet 2011;377:127–38. 21183212 10.1016/S0140-6736(10)62231-3 PMC3018568

[16] Coleman MP , Quaresma M , Berrino F , et al. Cancer survival in five continents: a worldwide population‐based study (CONCORD). Lancet Oncol 2008;9:730–56. 18639491 10.1016/S1470-2045(08)70179-7

[17] Allemani C , Weir HK , Carreira H , et al. Global surveillance of cancer survival 1995‐2009: analysis of individual data for 25 676 887 patients from 279 population‐based registries in 67 countries (CONCORD‐2). Lancet 2015;385:977–1010. 25467588 10.1016/S0140-6736(14)62038-9 PMC4588097

[18] Feinstein AR , Sosin DM , Wells CK. The Will Rogers phenomenon: stage migration and new diagnostic techniques as a source of misleading statistics for survival in cancer. N Engl J Med 1985;312:1604–8. 4000199 10.1056/NEJM198506203122504

[19] Cancer in NSW Aboriginal peoples: Incidence, mortality and survival. Cancer Institute NSW, 2012.

[20] Currow DT , Thomson W. Cancer in New South Wales: Incidence Report 2009. Cancer Institute NSW, February 2012.

[21] Morris M , Woods LM , Rogers N , et al. Ethnicity, deprivation and screening: survival from breast cancer among screening‐eligible women in the West Midlands diagnosed from 1989 to 2011. Br J Cancer 2015;113:548–55. 26079301 10.1038/bjc.2015.204 PMC4522622

[22] Maringe C , Li R , Mangtani P , et al. Cancer survival differences between South Asians and non‐South Asians of England in 1986‐2004, accounting for age at diagnosis and deprivation. Br J Cancer 2015;113:173–81. 26079299 10.1038/bjc.2015.182 PMC4647525

[23] Woods LM , Rachet B , Coleman MP. Choice of geographic unit influences socioeconomic inequalities in breast cancer survival. Br J Cancer 2005;92:1279–82. 15798765 10.1038/sj.bjc.6602506 PMC2361971

[24] Woods LM. International differences in breast cancer survival and 'cure' by social deprivation: a comparative study of England and Australia. PhD thesis: London: London School of Hygiene and Tropical Medicine, 2006.

[25] Forbes LJ , Simon AE , Warburton F , et al. Differences in cancer awareness and beliefs between Australia, Canada, Denmark, Norway, Sweden and the UK (the International Cancer Benchmarking Partnership): do they contribute to differences in cancer survival? Br J Cancer 2013;108:292–300. 23370208 10.1038/bjc.2012.542 PMC3566814

[26] Lejeune C , Sassi F , Ellis L , et al. Socio‐economic disparities in access to treatment and their impact on colorectal cancer survival. Int J Epidemiol 2010;39:710–7. 20378687 10.1093/ije/dyq048

[27] Morris E , Quirke P , Thomas JD , et al. Unacceptable variation in abdominoperineal excision rates for rectal cancer: time to intervene? Gut 2008;57:1690–7. 18535029 10.1136/gut.2007.137877

[28] Roberts MC , Wheeler SB , Reeder‐Hayes K. Racial/Ethnic and socioeconomic disparities in endocrine therapy adherence in breast cancer: a systematic review. Am J Public Health 2015;105 Suppl 3:e4–e15. 25905855 10.2105/AJPH.2014.302490 PMC4455526

[29] van Ryn M , Burke J. The effect of patient race and socio‐economic status on physicians' perceptions of patients. Soc Sci Med 2000;50:813–28. 10695979 10.1016/s0277-9536(99)00338-x

[30] Wheeler SB , Reeder‐Hayes KE , Carey LA. Disparities in breast cancer treatment and outcomes: biological, social, and health system determinants and opportunities for research. Oncologist 2013;18:986–93. 23939284 10.1634/theoncologist.2013-0243 PMC3780646

[31] McKenzie F , Ives A , Jeffreys M. Socio‐economic inequalities in survival from screen‐detected breast cancer in South West England: population‐based cohort study. Eur J Public Health 2012;22:41822: 10.1093/eurpub/ckr107 21891789

[32] Pacelli B , Carretta E , Spadea T , et al. Does breast cancer screening level health inequalities out? A population‐based study in an Italian region. Eur J Public Health 2014;24:280–5. 24008553 10.1093/eurpub/ckt119

[33] Puliti D , Miccinesi G , Manneschi G , et al. Does an organised screening programme reduce the inequalities in breast cancer survival? Ann Oncol 2012;23:319–23. 21515663 10.1093/annonc/mdr121

[34] Louwman WJ , van de Poll‐Franse LV , Fracheboud F , et al, Impact of a programme of mass mammography screening for breast cancer on socio‐economic variation in survival: a population‐based study. Breast Cancer Res Treat 2007;105:369–75. 17211536 10.1007/s10549-006-9464-9 PMC2190785

